# Associations between multiple perinatal exposures and risk of childhood hospitalisation with infection: a registry-based study in two countries

**DOI:** 10.1007/s10654-025-01266-1

**Published:** 2025-08-13

**Authors:** Isobel M. F. Todd, Lars Henning Pedersen, Maria C. Magnus, Natasha Nassar, Jessica E. Miller, David P. Burgner

**Affiliations:** 1 Department of Paediatrics, Royal Children’s Hospital, The University of Melbourne, 50 Flemington Rd, Parkville, VIC 3052 Australia; 2 Murdoch Children’s Research Institute, Parkville, VIC Australia; 3 Clinical Medicine, Aarhus University, Aarhus, Denmark; 4 Department of Obstetrics and Gynecology, Aarhus University Hospital, Aarhus, Denmark; 5 Centre for Fertility and Health, Norwegian Institute of Public Health, Oslo, Norway; 6 Child Population and Translational Health Research, Children’s Hospital Westmead Clinical School, Faculty of Medicine and Health, The University of Sydney, Camperdown, NSW Australia; 7 Leeder Centre for Health Policy, Economics and Data, Faculty of Medicine and Health, The University of Sydney, Camperdown, NSW Australia; 8 Charles Perkins Centre, The University of Sydney, Camperdown, NSW Australia; 9 Department of Infectious Diseases, Royal Children’s Hospital Melbourne, Parkville, VIC Australia; 10 Faculty of Health, Deakin University, Burwood, VIC Australia

**Keywords:** Pregnancy, Perinatal, Infection, Paediatric, Hospitalisation

## Abstract

Several perinatal exposures impact severe infection risk in offspring. However, most studies consider exposures in isolation rather than the impact of multiple co-occurring perinatal exposures. Here, we investigated the risk of hospitalised childhood infections associated with combinations of multiple perinatal exposures. We studied liveborn, singleton births in Denmark between 1997 and 2019 (*n* = 1,113,708) and Norway between 2008 and 2018 (*n* = 470,270) through national registry linkage. Seven perinatal exposures were evaluated including maternal antibiotic use during pregnancy, maternal smoking, maternal diabetes, hypertensive disorders of pregnancy, caesarean section birth, offspring being small for gestational age (SGA), and preterm birth. The outcome was hospitalised infections before 5 years age. Using Cox regression, we estimated hazard ratios (HR) of hospitalised infections for: (1) each exposure individually; (2) the cumulative number of exposures; (3) each unique combination of the seven exposures; (4) pairwise interactions between exposures. Results were combined through meta-analysis. Each exposure was individually associated with greater hospitalised infection risk with pooled HR estimates ranging from 1.08 (95% CI 1.08–1.09) for SGA to 1.45 (95% CI 1.43–1.46) for preterm birth. Cumulative risk was observed for increasing number of exposures from a pooled HR of 1.17 (95% CI 1.17–1.18) for one exposure to 1.88 (95% CI 1.78–1.99) for five or more exposures. Analyses of unique combinations of the exposures showed a particularly increased risk among preterm children with one or more additional exposures. Our findings can inform targeted strategies for the prevention and management of childhood infections. We highlight a simple, intuitive method for measuring risk associated with co-occurring exposures.

**Supplementary Information:**

The online version contains supplementary material available at 10.1007/s10654-025-01266-1.

## Introduction

Infections cause significant health burden in early childhood [1–3]. Despite extensive research on both genetic and environmental risk factors for infection, there remains substantial unexplained variation in childhood infection susceptibility and severity.

Infection burden peaks in the first few years of life and consequently exposures during pregnancy and around birth have been a focus of research exploring differential infection susceptibility and severity in children. Several exposures, including maternal smoking [4–6], prenatal antibiotic exposure [7–9], caesarean section birth [10], shorter gestational age [11–13], and lower birth weight [12–14] have each been associated with greater risk of overall and clinical groups of hospitalised infections. Despite the interrelatedness of these exposures and their frequent co-occurrence clinically, most studies have investigated either a single or a small number of exposures of interest, with related factors often adjusted for as confounding factors. Few studies evaluate the potential role of multiple adverse exposures during the perinatal period, which better reflects real-world experience. This limits clinical translation as it is unknown whether children with multiple adverse exposures or specific combinations of exposures have a particularly high risk of infections during the first years of life. Better risk stratification would allow prioritisation of targeted early childhood infection prevention and management.

In this study, our aim was to evaluate the risk of hospitalisation for infections during the first years of life associated with multiple inter-related perinatal exposures and thereby identify combinations of perinatal exposures which confer a particular increased infection risk for children.

## Methods

### Study population

Two separate registry-based cohorts were assembled from Norwegian and Danish administrative health data. The cohorts were identified from the Medical Birth Register of Norway and Denmark and included all liveborn singleton children born from 1 st January 2008 through 31 st December 2017 in Norway and from 1 st November 1997 through 31 st December 2018 in Denmark. We excluded multiple births, children with gestational age < 24 or > 43 weeks, birth weight < 500 or > 5500 g, children with congenital malformations recorded in the birth registries, and pregnancies where information on maternal smoking was not available (Denmark *n* = 26738, Norway *n* = 67816). In the Norwegian data, emigration dates were not available, and therefore, we additionally excluded children who emigrated from the study population (*n* = 15898). In the Danish data, maternal emigration dates were used as a proxy for censoring due to emigration. Birth records were linked to national hospital and prescription registries for both countries in the relevant periods.

### Exposures

We included seven perinatal exposures for which there was prior evidence of association with infection in offspring or relatedness with other exposures [4–15]. Start of pregnancy was calculated as the birth date minus the estimated gestational age, which is usually based on ultrasound estimates. For each exposure, a binary variable was created as follows: maternal dispensed prescriptions for antibiotics during pregnancy (any/none), maternal smoking during pregnancy (any/none), maternal diabetes (pre-gestational type 1 or 2 or gestational diabetes versus no diabetes), hypertensive disorders during pregnancy (any of pre-pregnancy hypertension, gestational hypertension, pre-eclampsia, eclampsia, HELLP versus none), mode of birth (caesarean versus vaginal), preterm birth (< 37 versus ≥37 weeks gestational age), and small for gestational age (SGA) (≤10th percentile versus > 10th percentile of birthweight for specific gestational age and sex). Further details on the diagnosis/procedure/prescription codes and registries used to create each exposure variable is presented in Supplementary Table 1.

### Outcomes

The primary outcome was infection-related hospitalisation, defined as any inpatient admission in the hospital registry with a primary or secondary diagnosis corresponding to our pre-specified set of diagnosis codes under the *International Classification of Diseases Tenth Revision* (ICD-10). We also excluded birth-related infections, which may reflect vertical transmission of pathogens in pregnancy or around the time of birth. In Danish analyses we excluded infection-related admissions that occurred before the hospital discharge date for the birth admission whereas in Norwegian analyses we excluded infections in the first 14 days of life as the hospital discharge date was unavailable. In our analysis, we considered the potential for more than one hospital admission with infection. Re-admissions within seven days of the previous admission’s discharge date were considered as the same infection event.

### Statistical analysis

Associations between perinatal exposures and infection-related hospitalisation were assessed using hazard ratios estimated from Cox proportional hazards regression models with child age as the time scale. This model was a Prentice–Williams–Peterson recurrent events model considering up to three separate infection-related hospitalisations [16]. The Prentice–Williams–Peterson model stratifies by event number (e.g., first, second, third hospitalised infection), estimating a different baseline hazard for each event number. This allows for the risk to vary according to event number which is plausible for our outcome of hospitalised infections. We used the total-time method of the Prentice–Williams–Peterson model. For our main analysis, we included infection-related hospitalisations occurring before 5 years of age to capture the peak period of infection-related hospitalisations in children. Censoring events in the analysis were death, emigration (available in Denmark only) or the end of follow-up (5 years of age or end of the study period, whichever occurred first). Two sensitivity analyses were conducted in which we evaluated all infections the first 2 years and 10 years of life, respectively. Exclusions due to missing exposure data (apart from pregnancy smoking) were minimal.

To evaluate the risks associated with the seven exposures individually and in combinations, we used four separate analytic approaches, basing our methodological approach on that of Nielsen et al. which also considered multiple co-occurring exposures [17]. All four approaches used the regression model as described above and included adjustment for background maternal and perinatal factors that were considered potential confounders, as identified from our causal directed acyclic graph (Supplementary Fig. 1): (1) maternal age, (2) parity, (3) year of birth, (4) season of birth, and as a proxy for socio-economic status (5a) maternal employment status (Denmark only), and (5b) maternal country of birth (Norway only). First, we considered the individual associations between each perinatal exposure and infection-related hospitalisation. Second, we examined the association between the cumulative number of adverse perinatal exposures (1, 2, 3, 4, 5+), where the group with no exposures was the reference category. Third, we estimated the association for each unique combination of the seven perinatal exposures, again where the group with no exposures were the reference category. This analysis was limited to exposure combinations with at least 100 children. In each analysis, separate estimates were generated for the Norwegian and Danish cohorts and these estimates were subsequently combined using fixed-effects meta-analysis. We chose a fixed-effects model as the two populations were relative similar in terms of data sources and the individual estimates. Finally, to examine possible interaction between exposures, we estimated interaction terms on the multiplicative and additive scales for each pairwise combination of perinatal exposures. For the multiplicative scale we report the interaction term as the ratio of hazard ratios (HR_11_/HR_01_HR_10_) and for the additive scale we report the relative excess risk due to interaction (RERI) measure calculated using the InteractionR package [18]. We tested interactions on both scales as each may provide different insight into the joint effects of exposures [19], however we focused our main results to interaction terms on the multiplicative scale in line with our other analyses. The multiplicative interaction term indicates whether the effect of two exposures present together is greater (interaction term > 1) or lesser (interaction term < 1) than the expected joint effect calculated from the product of their individual effects.

## Results

### Study population characteristics

Our analyses included 1,113,708 singleton children born in Denmark between 1997 and 2019 and 470,270 born in Norway between 2008 and 2018, giving a total analytical sample of 1,583,978. A total of 23.6% and 21.5% of the study population in Denmark and Norway respectively had one or more infection-related hospitalisation before the age of five. The distribution of maternal and perinatal characteristics for the two countries are shown in Table 1. The proportion of children who had each perinatal exposure of interest were similar between the two countries, albeit with somewhat higher maternal antibiotic prescriptions during pregnancy in the Norwegian study population (27.3% vs. 24.4%, respectively), and a higher proportion of smoking during pregnancy in the Danish study population (15.6% vs. 8.9%, respectively).

### Individual exposure associations with infection-related hospitalisation

**Fig. 1:**
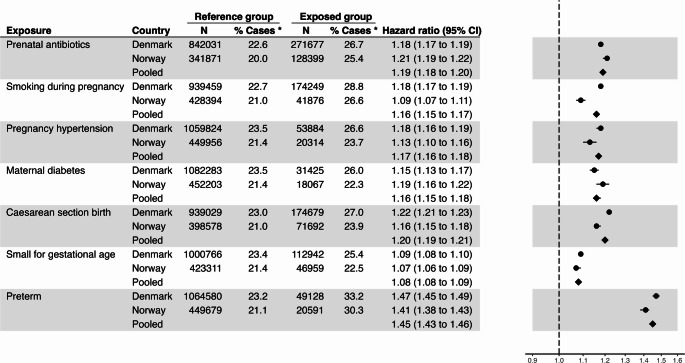
Risk of hospitalised infection for each exposure individually. * % Cases = the percentage of the exposure group who had one or more infection-related hospitalisation

Adjusted estimates of the individual risk for each exposure are shown in Fig. 1. All seven perinatal and pregnancy exposures were each associated with increased risk of infection-related hospitalisation. Similar estimates were observed across the two countries. In meta-analyses of hazard ratios across the two populations, the magnitude of risk was smallest for SGA (aHR 1.08, 95% CI 1.08–1.09); relatively similar across smoking during pregnancy (aHR 1.16, 95% CI 1.15–1.17), maternal diabetes (aHR 1.16, 95% CI 1.15–1.18), hypertensive disorders of pregnancy (aHR 1.17, 95% CI 1.16–1.18), prenatal antibiotics (aHR 1.19, 95% CI 1.18–1.20), and caesarean section (aHR 1.20, 95% CI 1.19–1.21); and highest in magnitude for preterm birth (aHR 1.45, 95% CI 1.43–1.46).

### Cumulative number of exposures

**Fig. 2:**
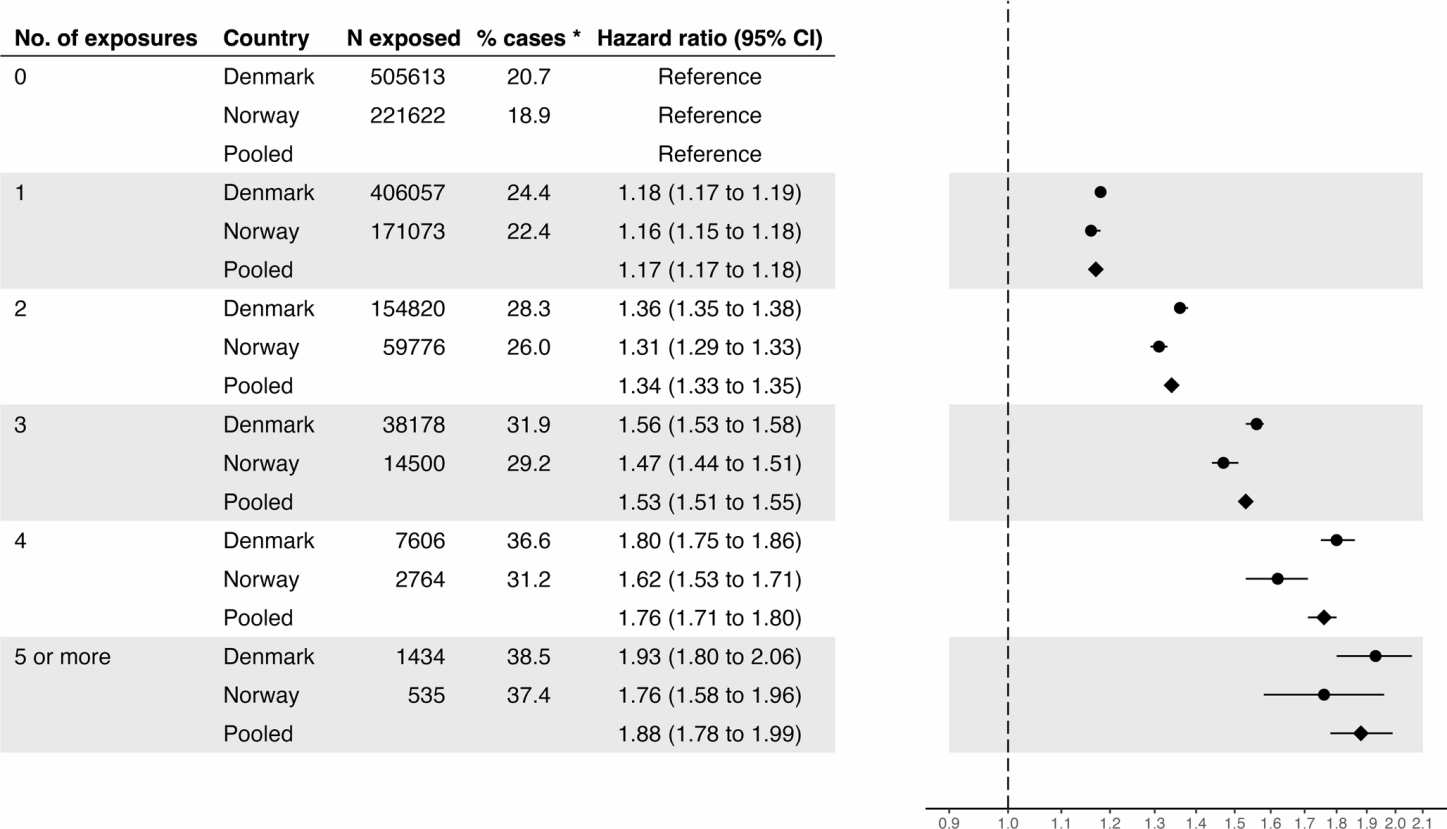
Risk of hospitalised infection by number of exposures. * % Cases = the percentage of the exposure group who had one or more infection-related hospitalisation.

Figure 2 shows the adjusted estimates from the analysis of infection-related hospitalisation according to the number of cumulative exposures. In the pooled analysis, a dose-response pattern was observed with the number exposures; the adjusted hazard ratio for hospitalised infections was 1.17 (95% CI 1.17 to 1.18) for one exposure compared to no exposures, and 1.88 (95% CI 1.78 to 1.99) for five or more exposures.

### Unique combinations of exposures

The adjusted estimates for the association between each unique combination of the seven exposures and hospitalised infections are shown in Table 2. A total of 86 and 68 unique exposure combinations with 100 or more exposed children were analysed for Denmark and Norway respectively. Nearly all combinations of exposures were associated with increased risk of hospitalised infections. The results showed similar patterns to the analyses of individual exposures, where combinations including preterm birth constituted the greatest risk for hospitalised infection, with estimates ranging from 1.43 (95% CI 1.31–1.56) for preterm children also exposed to maternal diabetes, to 2.14 (95% CI 1.93–2.38) for preterm children also exposed to prenatal antibiotics, caesarean section, and born small for gestational age. The four highest risk combinations in meta-analyses were children with prenatal antibiotic exposure, born preterm and by caesarean section in combination with another exposure. In combinations of exposures not involving preterm birth, the largest hazard ratios were approximately 1.6 and included four-way combinations of the most common exposures (prenatal antibiotic exposure and caesarean section) and two other exposures.

### Interactions between exposures

Table 3 shows the multiplicative interaction terms estimated for each pairwise combination of exposures and Supplementary Table 5 shows the full interaction analyses. The most notable interaction terms– where we observed similar results across both countries and the 95% confidence intervals for the interaction term were not inclusive of the null– were: (1) maternal diabetes together with pregnancy hypertension, and (2) maternal diabetes together with preterm birth, where the risk associated with the exposures together was less than what would be expected from their individual risks on the multiplicative scale; (3) caesarean section together with preterm birth on the additive and multiplicative scale; and (4) SGA together with preterm birth on the additive scale, where the combined risks were greater than that expected from their individual risks.

### Sensitivity analyses

Supplementary Tables 2–4 shows the results from sensitivity analyses where follow-up was until 2 years or 10 years of age rather than 5 years, which was the pre-specified period in the main analysis. The magnitude of risk tended to be slightly higher in analyses where follow-up was until 2 years and slightly lower in those until 10 years, but similar overall patterns to those in the main analyses were observed.

## Discussion

In this population-based study using administrative health data from two countries, we observed associations between seven putative adverse perinatal exposures with greater risk of hospitalisation for infections in childhood. There was a dose-response relationship, with the risk increasing with more co-occurring perinatal exposures. In analyses of unique combinations of exposures, preterm birth consistently showed the strongest association with the risk of hospitalised infection, with preterm children who also had additional perinatal exposures constituting a particularly vulnerable group. This study is the first to consider the combined risks associated with these seven co-occurring and interrelated perinatal exposures.

Our findings are consistent with previous studies indicating an increased risk of childhood infections among children exposed to prenatal smoking or antibiotics, and children born by caesarean section, at shorter gestational age, or with lower birthweight [4–13]. These previous studies have showed further detail such as trimester-specific and dose-response effects for prenatal antibiotic exposure [7, 9], separate effects for emergency and elective caesarean section [10], and the variation in risk across ranges of gestational age and birthweight [11–13]. However, most previous studies have only considered single exposures. We also found associations between hypertensive disorders of pregnancy and maternal diabetes with infections in children; common exposures for which there are limited data [20].

We found that preterm children constitute a particularly vulnerable group, and that preterm children who are also exposed to prenatal antibiotics, caesarean section, and one or more other exposures have a much higher risk of being hospitalised for infections compared to unexposed. Interaction analyses showed both positive additive and multiplicative interactions for caesarean section and SGA in combination with preterm birth. This is in keeping with previous studies showing a particularly heightened risk of hospitalised infections for children born both SGA and preterm [13] and those born preterm by caesarean section [21].

Many of the exposures in this study will serve as proxy measures for one or more physiological and clinical factors that could impact infection risk. There are several potential mechanisms underlying the observed associations between perinatal exposures and infections. For example, children born preterm have sub-optimal immune responses and lower protection from gestation-dependent transplacental transfer of maternal antibodies [22]. In addition, in the preterm population, immature lung development and its sequelae increase the risk of respiratory infections, which may be compounded by the effects of maternal smoking during pregnancy on infant lung function [23]. Pregnancy antibiotic exposure, caesarean section, and preterm birth may influence the composition of the infant gut microbiome, which is thought to impact immune development [24–27]. Clinical interventions such as antenatal corticosteroids and intrapartum antibiotics may impact infection risk and are more common in pregnancies with hypertension, and in caesarean section and/or preterm births [28–30]. Upstream factors such as infections during pregnancy which lead to antibiotic use, or maternal overweight/obesity preceding hypertension, diabetes, or caesarean section are also relevant [31–34]. The exposures investigated here are common and highly interrelated [15]. Mechanistic studies informed by these epidemiological data will improve understanding of these pathways and identify early life targets for intervention.

A significant strength of this study was the use of total population data across two countries. Total population data allows for a sufficiently large sample to study multiple exposures (including those that were less common) and rare combinations of exposures with reasonable precision. For example, we were able to examine multiple combinations of four and five exposures, where the prevalence of the exposure combination in the population was as low as 0.01%. Second, bias related to study participation or loss to follow up are minimised. Third, the comparison of findings from two settings and the consistency of the estimates give greater confidence in the study findings. Across our analyses, the direction of risk was consistent in the two countries and the exposure combinations which stood out as having a higher magnitude of risk tended to be the same. Only one combination of exposures (hypertension, diabetes and SGA in Denmark) resulted in a hazard ratio below 1, but this likely reflects a small exposure group and the large number of exposure combinations tested. In both countries, hospital care is free and so the outcome of hospitalised infections is a relatively unbiased measure of infection severity. We acknowledge that our measured associations do not represent exact estimates of causal effects. However, we believe our approach is a pragmatic and intuitive method for assessing the combined risks associated with multiple exposures for a disease outcome, which represents the reality of frequently co-occurring exposures.

There are some important limitations that should be noted. First, we were not able to account for the effect of postnatal exposures, which may act as mediators or effect modifiers. For example, breastfeeding rates are generally lower in children born by caesarean section and breastfeeding is protective against infection [35, 36]. Although, the proportion of children who are breastfed is high in both Norway and Denmark [37]. Maternal smoking during pregnancy will correlate with postnatal household tobacco smoke exposure, which is not captured by registry data. Second, the binary categorisation of the exposures will represent an averaging of risks which may be heterogenous within a category. For example, risks may differ for light versus heavy maternal smoking; for different types of diabetes, hypertensive disorders, or caesarean section; and for prenatal antibiotic dose, timing and class. Third, while we found similar results in Denmark and Norway, the wider generalisability of the findings warrants replication in other populations. In other settings, the frequency of key exposures, such as preterm birth and caesarean section birth are generally higher than those observed here [38, 39]. More broadly, the nature of the data directs which factors can be studied and how they are measured. The sensitivity of the registry data should also be considered. For some of our exposures (mode of birth, gestational age, birthweight) the data is likely to be accurate, whereas for other maternal conditions (e.g., hypertensive disorders) sensitivity may be lower [40]. Given its inherent stigma, self-reported smoking during pregnancy would be anticipated to have the most inaccuracy of measurement. Notwithstanding, smoking exposure misclassification would likely be non-differential and therefore bias the associations in our study towards the null and underestimate the true associations.

There are several important clinical, public health, and research implications of these findings. Our identification of particular high risk exposure combinations may inform clinical management based on risk stratification and priority populations for preventive interventions (e.g., timely and complete vaccination and more aggressive early management of infections in high-risk groups). Our findings also lend weight to public health efforts to address specific modifiable exposures and their determinants (e.g. smoking reduction, lifestyle behaviours associated with development of diabetes and hypertension, and inappropriate use of antibiotics in pregnancy). From a research perspective, we have highlighted hypertensive disorders of pregnancy and maternal diabetes as potential novel risk factors for infections in offspring that should be further explored, particularly given their increasing incidence [41]. We have also highlighted a methodological approach for studying the combined risks for co-occurring exposures, which is the usual scenario in real world settings. Our approach demonstrates a simple and intuitive method for visualising combined risk associated with co-occurring exposures for a single outcome and is readily transferable to other disease outcomes with co-occurring exposures.

In conclusion, our findings show a cumulative increased risk of childhood hospitalised infections with multiple adverse perinatal exposures. Children born preterm with additional adverse exposures are at a particular increased risk regardless of the additional exposures. Findings can inform targeted strategies for prevention and management of infection in early childhood.

**Table 1:** Maternal and child characteristics across the two countries

Characteristic	Denmark (*N* = 1 113 708)	Norway (*N* = 470 270)
Prenatal antibiotics, n (%)	271 677 (24.4)	128 399 (27.3)
Smoking during pregnancy, n (%)	174 249 (15.6)	41 876 (8.9)
Pregnancy hypertension, n (%)	53 884 (4.8)	20 314 (4.3)
Diabetes mellitus, n (%)	31 425 (2.8)	18 067 (3.8)
Caesarean section, n (%)	174 679 (15.7)	71 692 (15.2)
Small for gestational age, n (%)	112 942 (10.1)	46 959 (10.0)
Preterm birth, n (%)	49 128 (4.4)	20 591 (4.4)
Maternal age		
< 20	14 782 (1.3)	8123 (1.7)
20–24	125 757 (11.3)	64 689 (13.8)
25–29	373 398 (33.5)	152 264 (32.4)
30–34	394 716 (35.4)	155 111 (33.0)
35–39	173 163 (15.5)	75 009 (16.0)
40+	31 892 (2.9)	15 074 (3.2)
Parity, n (%)		
0	488 035 (43.8)	197 322 (42.0)
1	410 438 (36.9)	172 495 (36.7)
2	150 518 (13.5)	72 531 (15.4)
≥ 3	52 746 (4.7)	27 922 (5.9)
Missing	11 971 (1.1)	
Maternal income (Denmark)		
In education	66 472 (6.0)	N/A
Retiree	6342 (0.6)	N/A
Self-employed or working spouse	24 181 (2.2)	N/A
Transfer payment	264 304 (23.7)	N/A
Unemployed	17 781 (1.6)	N/A
Wage earner	568 912 (51.1)	N/A
Wage earner - management level	165 240 (14.8)	N/A
Missing	476 (0.0)	N/A
Maternal country of birth (Norway)		
High income	N/A	381 471 (81.1)
Central Eastern Europe and Central Asia	N/A	30 920 (6.6)
Low and middle income	N/A	53 914 (11.5)
Missing	N/A	3965 (0.8)
Birthweight (g), mean (SD)	3531 (533)	3542 (534)
Child sex female, n (%)	547 193 (49.1)	229 839 (48.9)
Hospitalised infection, *n* (%)		
0	850 689 (76.4)	369 260 (78.5)
1	189 644 (17.0)	67 601 (14.4)
2	47 480 (4.3)	19 235 (4.1)
≥3	25 895 (2.3)	14 174 (3.0)

SD, standard deviation; N/A, not applicable

**Table 2:**
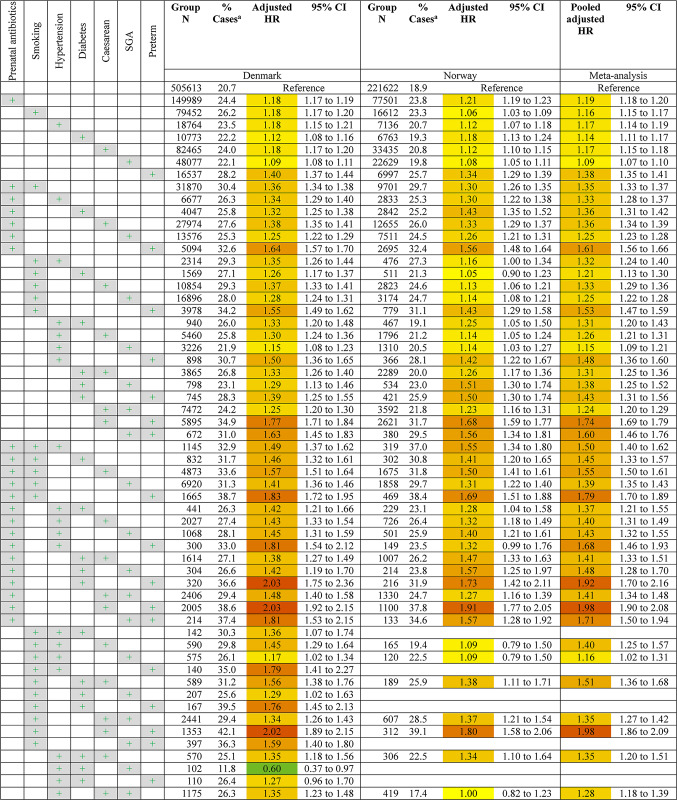

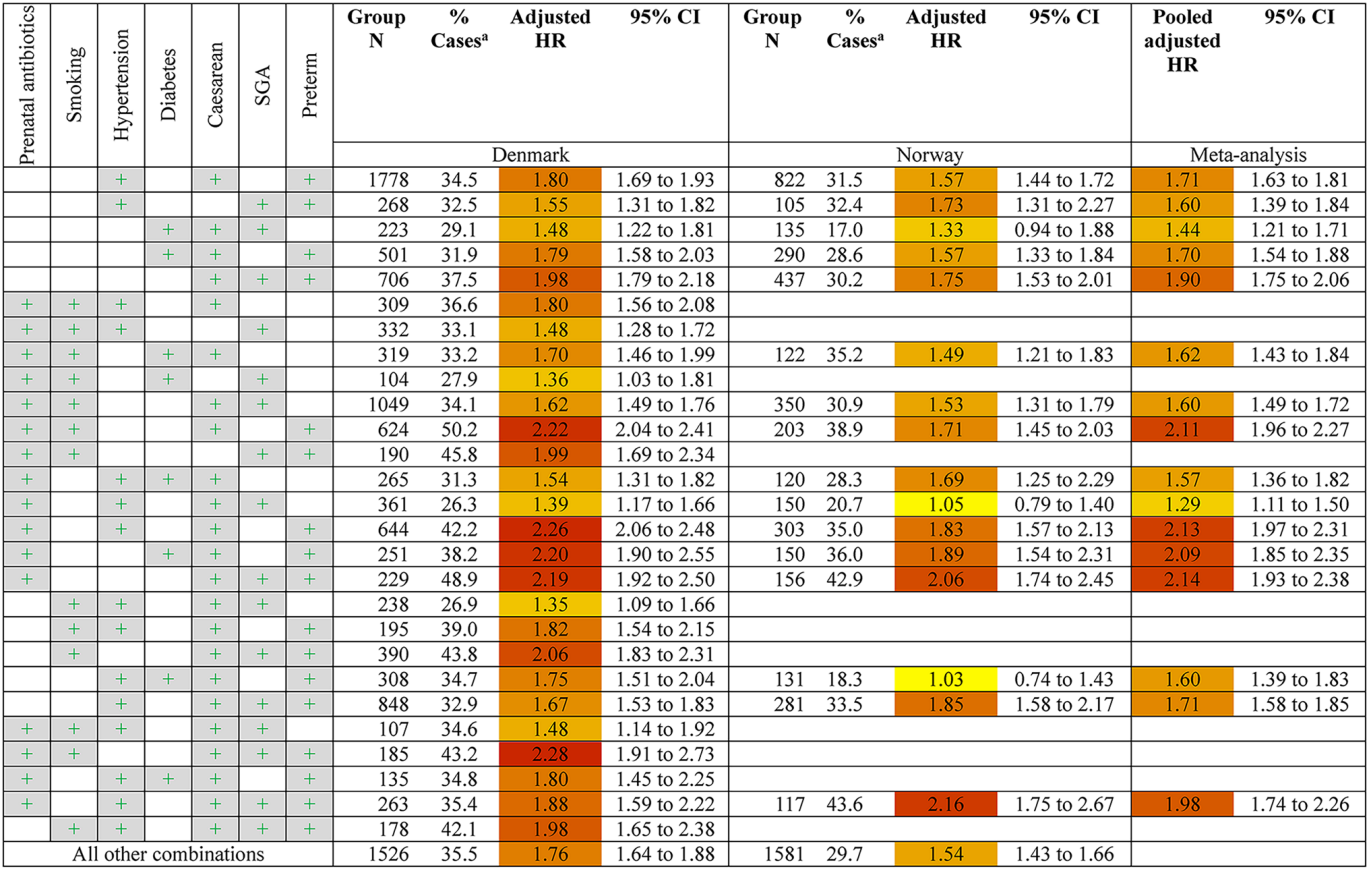
Risk of hospitalised infection by combination of exposures

^a^ % Cases = the percentage of the exposure group who had one or more infection-related hospitalisation

**Table 3:** Interaction terms for pairwise exposure combinations

Pairwise exposure combination	Multiplicative interaction term (95% CI)	
	Denmark	Norway
Prenatal antibiotics and maternal smoking	0.98 (0.96–0.99)	1.02 (0.99–1.05)
Prenatal antibiotics and pregnancy hypertension	0.97 (0.94–1.00)	0.96 (0.91–1.01)
Prenatal antibiotics and maternal diabetes	0.98 (0.94–1.02)	0.98 (0.93–1.04)
Prenatal antibiotics and caesarean section	0.99 (0.97–1.01)	0.98 (0.95–1.01)
Prenatal antibiotics and SGA	0.98 (0.96–1.00)	0.95 (0.92–0.98)
Prenatal antibiotics and preterm	1.00 (0.97–1.03)	0.95 (0.91–0.99)
Maternal smoking and pregnancy hypertension	0.94 (0.91–0.98)	0.94 (0.87–1.02)
Maternal smoking and maternal diabetes	0.95 (0.91–1.00)	0.88 (0.81–0.96)
Maternal smoking and caesarean section	0.99 (0.97–1.01)	1.00 (0.96–1.04)
Maternal smoking and SGA	0.97 (0.95–0.99)	0.99 (0.95–1.03)
Maternal smoking and preterm	0.94 (0.92–0.97)	0.97 (0.91–1.02)
Pregnancy hypertension and maternal diabetes	0.94 (0.89–0.99)	0.90 (0.83–0.99)
Pregnancy hypertension and caesarean section	0.99 (0.96–1.02)	0.97 (0.92–1.02)
Pregnancy hypertension and SGA	0.93 (0.90–0.97)	1.00 (0.94–1.07)
Pregnancy hypertension and preterm	0.96 (0.92–0.99)	0.95 (0.90–1.02)
Maternal diabetes and caesarean section	0.98 (0.94–1.02)	0.93 (0.88–0.98)
Maternal diabetes and SGA	0.97 (0.90–1.04)	1.06 (0.95–1.17)
Maternal diabetes and preterm	0.93 (0.88–0.98)	0.88 (0.81–0.95)
Caesarean section and SGA	0.99 (0.97–1.02)	1.02 (0.98–1.06)
Caesarean section and preterm	1.07 (1.04–1.10)	1.07 (1.03–1.12)
SGA and preterm	1.03 (0.99–1.07)	1.10 (1.03–1.17)

## Electronic supplementary material

Below is the link to the electronic supplementary material.

Supplementary Material 1

## Data Availability

The datasets used to conduct the analyses presented in this article are not readily available because of data protection laws in Denmark and Norway. Requests to access the datasets should be made to Danish and Norwegian health authorities. Analysis code is available upon reasonable request.

## Declarations

### **Copyright statement**

For the purposes of open access, the author has applied a CC BY public copyright licence to any Author Accepted Manuscript version arising from this submission.
