# Supplementary material for: Associations between multiple perinatal exposures and risk of childhood hospitalisation with infection: a registry-based study in two countries
Source: Eur J Epidemiol. 2025 Aug 13;40(10):1231–41. doi: 10.1007/s10654-025-01266-1 (PMC12660341; doi:10.1007/s10654-025-01266-1)
Supplement: Supplementary file 1 — Supplementary Material 1 [file 10654_2025_1266_MOESM1_ESM.pdf]

Supplementary material

Supplementary Figure 1 - Causal directed acyclic graph

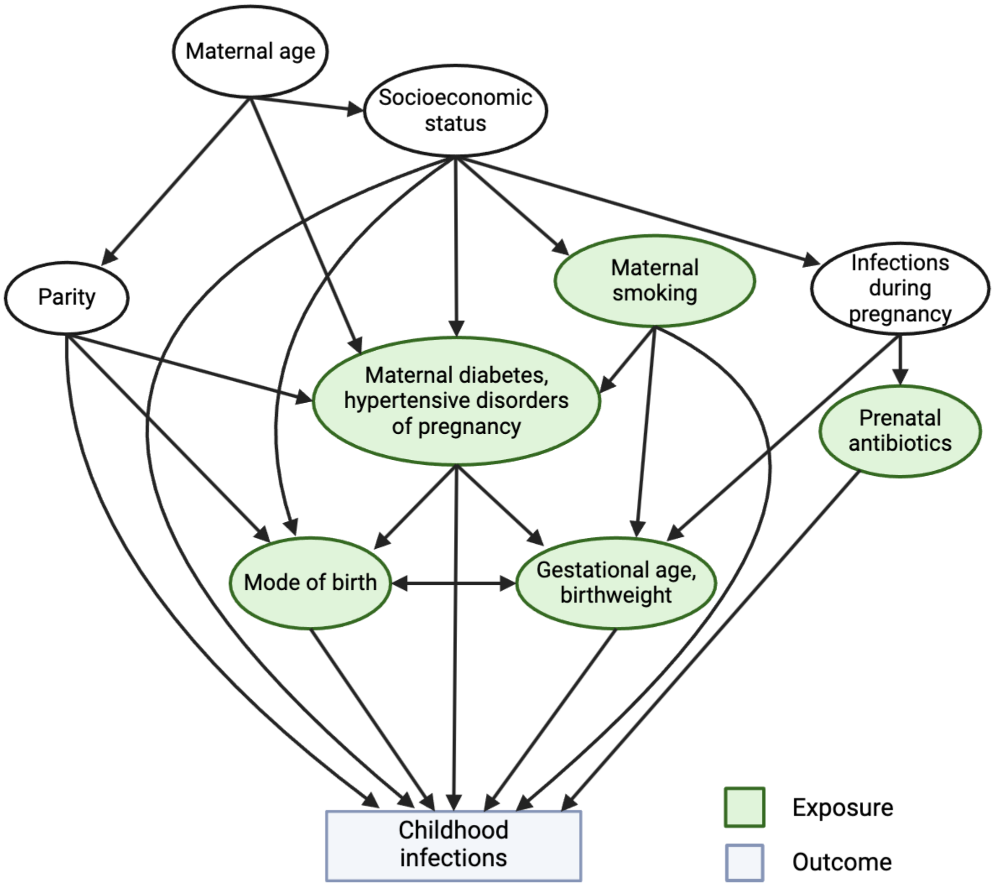

Supplementary Table 1 – Details on exposure variable data sources and coding

Registry	Registry variable/source	Ascertainment window	Medication, diagnosis or procedure code	Medication, diagnosis or procedure code description	Detailed variable	Binary variable
**Exposure = prenatal antibiotics**						
Norwegian Prescription Registry, Danish prescription registry	ATC code	Estimated pregnancy start date* – birth date	Beginning with J01	Antibacterials for systemic use	Prenatal antibiotic use	Prenatal antibiotic use
**Exposure = maternal smoking during pregnancy**						
Medical Birth Registry of Norway	Variable ROYK_BEG equal to "No" and variable ROYK_AVSL equal to "No" or is missing				No smoking	No smoking
	Variables ROYK_BEG or ROYK_AVSL equal to "Sometimes" or "Daily"				Smoking	Smoking
	Variable ROYK_BEG is missing				Missing smoking information – excluded from analyses	Missing smoking information – excluded from analyses
Danish National Patient Registry	Supplementary diagnoses		DUT00	Mother didn't smoke	No smoking	No smoking
			DUT10	Mother stopped smoking in the first trimester	Stopped smoking	Smoking
			DUT11	Mother stopped smoking after the 1st trimester	Stopped smoking	
			DUT20	Mother smokes up to 5 cigarettes daily	Smoking	
			DUT21	Mother smokes 6-10 cigarettes daily		
			DUT22	Mother smokes 11-20 cigarettes daily		
			DUT23	Mother smokes over 20 cigarettes daily		
			DUT29	Mother smokes, amount not stated		

Registry	Registry variable/source	Ascertainment window	Medication, diagnosis or procedure code	Medication, diagnosis or procedure code description	Detailed variable	Binary variable
			DUT99	Mother's smoking status not disclosed	Missing smoking information – excluded from analyses	Missing smoking information – excluded from analyses
**Exposure = hypertensive disorders of pregnancy**						
Medical Birth Registry of Norway	HYPERTENSJON_KRONISK				Pre-existing hypertension	Hypertensive disorders of pregnancy
	HYPERTENSJON_ALENE				Gestational hypertension	
	EKLAMPSI				Eclampsia	
	PREEKL				Pre-eclampsia	
	PREEKLTIDL				Pre-eclampsia	
	HELLP				HELLP syndrome	
Danish National Patient Registry	Diagnoses	Estimated pregnancy start date* – birth date + 7 days	DO10	Pregnancy, childbirth and maternity with complicating hypertension	Hypertension during pregnancy unspecified	Hypertensive disorders of pregnancy
			DO100	Pre-existing essential hypertension complicating pregnancy, childbirth and the puerperium	Pre-existing hypertension	
			DO101	Pre-existing hypertensive heart disease complicating pregnancy, childbirth and the puerperium		
			DO102	Pre-existing hypertensive renal disease complicating pregnancy, childbirth and the puerperium		
			DO103	Pre-existing hypertensive heart and renal disease complicating pregnancy, childbirth and the puerperium		
			DO104	Pre-existing secondary hypertension complicating pregnancy, childbirth and the puerperium		

Registry	Registry variable/source	Ascertainment window	Medication, diagnosis or procedure code	Medication, diagnosis or procedure code description	Detailed variable	Binary variable
			DO109	Unspecified pre-existing hypertension complicating pregnancy, childbirth and the puerperium		
			DO11	Pre-eclampsia superimposed on chronic hypertension	Pre-eclampsia	
			DO119 (A, B, C)		Pregnancy with complicating hypertension	
			DO13	Gestational [pregnancy-induced] hypertension	Gestational hypertension	
			DO139	Gestational hypertension without proteinuria		
			DO14	Pre-eclampsia	Pre-eclampsia	
			DO140	Mild to moderate pre-eclampsia		
			DO141	Severe pre-eclampsia		
			DO142	HELLP syndrome	HELLP syndrome	
			DO149	Pre-eclampsia, unspecified	Pre-eclampsia	
			DO15	Eclampsia	Eclampsia	
			DO150	Eclampsia in pregnancy		
			DO151	Eclampsia in labor		
			DO152	Eclampsia in the puerperium		
			DO159	Eclampsia, unspecified as to time period		
			DO16	Unspecified maternal hypertension	Hypertension during pregnancy unspecified	
			DO169	Unspecified maternal hypertension	Hypertension during pregnancy unspecified	
**Exposure = maternal type 2 and gestational diabetes**						
Medical Birth Registry of Norway	DIABETES_MELITTUS equal to "Pregestational diabetes – Type 1"				Type 1 diabetes	Diabetes

Registry	Registry variable/source	Ascertainment window	Medication, diagnosis or procedure code	Medication, diagnosis or procedure code description	Detailed variable	Binary variable
	DIABETES_MELITTUS equal to "Pregestational diabetes – Type 2"				Type 2 diabetes	
	DIABETES_MELITTUS equal to "Pregestational diabetes – Unspecified / other"				Pre-existing diabetes unspecified	
	DIABETES_MELITTUS equal to "Gestational diabetes"				Gestational diabetes	
	DIABETES_MELITTUS equal to "Antidiabetic medication during pregnancy registered without further information"				Diabetes unspecified	
Danish National Patient Registry	Diagnoses	Estimated pregnancy start date* – birth date + 7 days	DO24	Diabetes during pregnancy, birth and maternity	Diabetes unspecified	Diabetes
			DO240 (A, B, C)	Type 1 diabetes during pregnancy, birth, or maternity	Type 1 diabetes	
			DO241 (A, B, C)	Type 2 diabetes during pregnancy, birth, or maternity	Type 2 diabetes	
			DO242 (A, B, C)	Pre-existing malnutrition-related diabetes		
			DO243 (A, B, C)	Pre-existing diabetes during pregnancy type unspecified	Pre-existing diabetes unspecified	
			DO244 (B, C, D, E)	Gestational diabetes during pregnancy, birth, or maternity	Gestational diabetes	
			DO245	Newly discovered diabetes during pregnancy	Diabetes unspecified	
			DO249	Diabetes during pregnancy type unspecified		
**Exposure = caesarean section**						
Medical Birth Registry of Norway	KSNITT				Caesarean section	Caesarean section

Registry	Registry variable/source	Ascertainment window	Medication, diagnosis or procedure code	Medication, diagnosis or procedure code description	Detailed variable	Binary variable
Danish National Patient Registry	Procedures	Birth date ± 7 days	KMCA10A	Caesarean section in the isthmus uterus acutely performed before birth	Caesarean section	Caesarean section
			KMCA10B	Caesarean section in the isthmus uterus performed planned before birth		
			KMCA10D	Caesarean section in the isthmus uterus during labour before planned caesarean section		
			KMCA10E	Caesarean section in the isthmus uterus during childbirth due to birth complications		
			NZTB10A	Notification of grade 1 caesarean section to the anesthesia department		
			NZTB10B	Notification of grade 2 caesarean section to the anesthesia department		
			NZTB10C	Notification of grade 3 caesarean section to the anesthesia department		

Supplementary Table 2 – Risk of hospitalised infection for each exposure individually in main analysis and sensitivity analyses

**Exposure**	**Crude HR (95% CI)**	**Adjusted HR (95% CI)**	**Crude HR (95% CI)**	**Adjusted HR (95% CI)**
	Denmark		Norway	
**Main analysis**				
Prenatal antibiotics	1.20 (1.19 to 1.21)	1.18 (1.17 to 1.19)	1.23 (1.21 to 1.24)	1.21 (1.19 to 1.22)
Smoking during pregnancy	1.24 (1.23 to 1.25)	1.18 (1.17 to 1.19)	1.14 (1.12 to 1.16)	1.09 (1.07 to 1.11)
Pregnancy hypertension	1.17 (1.15 to 1.19)	1.18 (1.16 to 1.19)	1.11 (1.08 to 1.14)	1.13 (1.10 to 1.16)
Maternal diabetes	1.16 (1.14 to 1.18)	1.15 (1.13 to 1.17)	1.14 (1.11 to 1.17)	1.19 (1.16 to 1.22)
Caesarean section birth	1.21 (1.20 to 1.22)	1.22 (1.21 to 1.23)	1.14 (1.12 to 1.15)	1.16 (1.15 to 1.18)
SGA	1.10 (1.09 to 1.11)	1.09 (1.08 to 1.10)	1.07 (1.05 to 1.09)	1.07 (1.06 to 1.09)
Preterm	1.47 (1.45 to 1.49)	1.47 (1.45 to 1.49)	1.40 (1.38 to 1.43)	1.41 (1.38 to 1.43)
**Sensitivity analysis – follow up ends at 2 years of age**				
Prenatal antibiotics	1.22 (1.21 to 1.23)	1.19 (1.18 to 1.20)	1.25 (1.24 to 1.27)	1.23 (1.21 to 1.24)
Smoking during pregnancy	1.27 (1.26 to 1.28)	1.22 (1.21 to 1.23)	1.12 (1.10 to 1.14)	1.06 (1.04 to 1.08)
Pregnancy hypertension	1.17 (1.16 to 1.19)	1.19 (1.17 to 1.21)	1.11 (1.08 to 1.14)	1.14 (1.10 to 1.17)
Maternal diabetes	1.17 (1.15 to 1.20)	1.15 (1.13 to 1.18)	1.15 (1.11 to 1.18)	1.21 (1.17 to 1.24)
Caesarean section birth	1.23 (1.22 to 1.24)	1.24 (1.23 to 1.25)	1.14 (1.12 to 1.16)	1.18 (1.16 to 1.20)
SGA	1.10 (1.08 to 1.11)	1.10 (1.09 to 1.11)	1.06 (1.04 to 1.08)	1.09 (1.06 to 1.11)
Preterm	1.52 (1.50 to 1.54)	1.54 (1.52 to 1.56)	1.49 (1.45 to 1.52)	1.50 (1.47 to 1.54)
**Sensitivity analysis – follow up ends at 10 years of age**				
Prenatal antibiotics	1.19 (1.18 to 1.20)	1.17 (1.16 to 1.18)	1.22 (1.20 to 1.23)	1.20 (1.19 to 1.21)
Smoking during pregnancy	1.21 (1.20 to 1.22)	1.16 (1.15 to 1.17)	1.14 (1.12 to 1.15)	1.09 (1.07 to 1.11)
Pregnancy hypertension	1.16 (1.14 to 1.18)	1.17 (1.15 to 1.18)	1.11 (1.09 to 1.14)	1.13 (1.10 to 1.15)
Maternal diabetes	1.15 (1.13 to 1.17)	1.14 (1.12 to 1.16)	1.14 (1.11 to 1.16)	1.18 (1.15 to 1.21)
Caesarean section birth	1.19 (1.18 to 1.20)	1.20 (1.19 to 1.21)	1.13 (1.11 to 1.14)	1.15 (1.14 to 1.17)
SGA	1.09 (1.08 to 1.10)	1.08 (1.07 to 1.09)	1.07 (1.05 to 1.09)	1.07 (1.05 to 1.09)
Preterm	1.43 (1.41 to 1.45)	1.43 (1.41 to 1.44)	1.37 (1.34 to 1.39)	1.37 (1.34 to 1.39)

Supplementary Table 3 – Risk of hospitalised infection by number of exposures in main analysis and sensitivity analyses

Number of exposures	Group N	Group n	Crude (95% CI)	Adjusted (95% CI)	Group N	Group n	Crude (95% CI)	Adjusted (95% CI)
	Denmark				Norway			
**Main analysis**								
0	505613	104422	Reference	Reference	221622	41870	Reference	Reference
1	406057	99259	1.19 (1.19 to 1.20)	1.18 (1.17 to 1.19)	171073	38306	1.17 (1.15 to 1.18)	1.16 (1.15 to 1.18)
2	154820	43803	1.40 (1.39 to 1.41)	1.36 (1.35 to 1.38)	59776	15539	1.32 (1.30 to 1.34)	1.31 (1.29 to 1.33)
3	38178	12196	1.60 (1.58 to 1.63)	1.56 (1.53 to 1.58)	14500	4233	1.48 (1.45 to 1.52)	1.47 (1.44 to 1.51)
4	7606	2787	1.84 (1.79 to 1.90)	1.80 (1.75 to 1.86)	2764	862	1.62 (1.54 to 1.71)	1.62 (1.53 to 1.71)
5 or more	1434	552	1.96 (1.84 to 2.10)	1.93 (1.80 to 2.06)	535	200	1.76 (1.59 to 1.96)	1.76 (1.58 to 1.96)
**Sensitivity analysis – follow up ends at 2 years of age**								
0	505613	81754	Reference	Reference	221622	31837	Reference	Reference
1	406057	78760	1.21 (1.20 to 1.22)	1.19 (1.18 to 1.20)	171073	29215	1.18 (1.16 to 1.19)	1.18 (1.16 to 1.19)
2	154820	35366	1.44 (1.42 to 1.45)	1.40 (1.39 to 1.42)	59776	11793	1.34 (1.32 to 1.37)	1.34 (1.32 to 1.37)
3	38178	9982	1.66 (1.63 to 1.69)	1.63 (1.60 to 1.66)	14500	3267	1.51 (1.47 to 1.56)	1.52 (1.48 to 1.57)
4	7606	2327	1.94 (1.88 to 2.01)	1.92 (1.86 to 1.98)	2764	669	1.65 (1.55 to 1.76)	1.67 (1.57 to 1.78)
5 or more	1434	468	2.15 (2.00 to 2.32)	2.15 (1.99 to 2.32)	535	162	1.89 (1.68 to 2.14)	1.92 (1.70 to 2.16)
**Sensitivity analysis – follow up ends at 10 years of age**								
0	505613	115195	Reference	Reference	221622	44861	Reference	Reference
1	406057	108080	1.18 (1.17 to 1.19)	1.17 (1.16 to 1.17)	171073	40877	1.16 (1.15 to 1.17)	1.15 (1.14 to 1.16)
2	154820	47284	1.37 (1.36 to 1.38)	1.34 (1.32 to 1.35)	59776	16544	1.31 (1.29 to 1.33)	1.30 (1.28 to 1.32)
3	38178	13030	1.55 (1.53 to 1.58)	1.51 (1.49 to 1.53)	14500	4505	1.47 (1.43 to 1.50)	1.45 (1.42 to 1.49)
4	7606	2936	1.77 (1.72 to 1.82)	1.73 (1.68 to 1.78)	2764	904	1.57 (1.49 to 1.65)	1.56 (1.48 to 1.64)
5 or more	1434	575	1.87 (1.76 to 2.00)	1.83 (1.72 to 1.96)	535	205	1.67 (1.50 to 1.85)	1.66 (1.49 to 1.84)

Supplementary Table 4 – Risk of hospitalised infection by combinations of exposures in sensitivity analyses

Prenatal antibiotics	Smoking	Hypertension	Diabetes	Caesarean	SGA	Preterm	**Group N**	**% cases**	**Adjusted (95% CI)**	**% cases**	**Adjusted (95% CI)**	**Group N**	**% cases**	**Adjusted (95% CI)**	**% cases**	**Adjusted (95% CI)**
							Denmark					Norway				
								Follow up to 2-years age		Follow up to 10-years age			Follow up to 2-years age		Follow up to 10-years age	
							505613	16.2	Reference	22.8	Reference	221622	14.4	Reference	18.9	Reference
+							149989	19.4	1.19 (1.17-1.20)	26.5	1.17 (1.16-1.18)	77501	18.4	1.23 (1.21-1.25)	23.8	1.21 (1.19-1.23)
	+						79452	20.6	1.21 (1.19-1.23)	28.8	1.16 (1.14-1.17)	16612	16.5	1.02 (0.99-1.06)	23.3	1.06 (1.03-1.09)
		+					18764	18.5	1.17 (1.14-1.21)	25.6	1.17 (1.14-1.20)	7136	15.4	1.15 (1.09-1.21)	20.7	1.12 (1.07-1.18)
			+				10773	17.9	1.13 (1.08-1.17)	23.8	1.12 (1.08-1.16)	6763	15.4	1.20 (1.14-1.27)	19.3	1.18 (1.13-1.24)
				+			82465	19.2	1.20 (1.18-1.21)	25.9	1.17 (1.15-1.18)	33435	15.9	1.13 (1.11-1.16)	20.8	1.12 (1.10-1.15)
					+		48077	17.2	1.10 (1.07-1.12)	24.2	1.09 (1.07-1.10)	22629	14.8	1.09 (1.06-1.13)	19.8	1.08 (1.05-1.11)
						+	16537	22.8	1.46 (1.42-1.50)	30.6	1.36 (1.33-1.40)	6997	20.8	1.45 (1.38-1.51)	25.7	1.34 (1.29-1.39)
+	+						31870	24.4	1.41 (1.38-1.44)	33.1	1.33 (1.31-1.35)	9701	21.8	1.29 (1.25-1.34)	29.7	1.30 (1.26-1.35)
+		+					6677	20.7	1.33 (1.27-1.39)	28.5	1.33 (1.28-1.38)	2833	19.2	1.34 (1.24-1.44)	25.3	1.30 (1.22-1.38)
+			+				4047	21.3	1.33 (1.25-1.41)	27.7	1.31 (1.24-1.37)	2842	21.0	1.51 (1.42-1.62)	25.2	1.43 (1.35-1.52)
+				+			27974	22.5	1.41 (1.38-1.44)	29.6	1.36 (1.33-1.39)	12655	20.0	1.36 (1.31-1.40)	26.0	1.33 (1.29-1.37)
+					+		13576	20.3	1.28 (1.24-1.32)	27.8	1.25 (1.21-1.28)	7511	18.9	1.33 (1.27-1.39)	24.5	1.26 (1.21-1.31)
+						+	5094	26.7	1.69 (1.61-1.77)	34.8	1.59 (1.53-1.65)	2695	25.8	1.69 (1.59-1.79)	32.4	1.56 (1.48-1.64)
	+	+					2314	23.5	1.42 (1.32-1.53)	31.9	1.33 (1.25-1.41)	476	18.3	1.11 (0.92-1.34)	27.3	1.16 (1.00-1.34)
	+		+				1569	21.5	1.28 (1.17-1.40)	28.7	1.22 (1.13-1.32)	511	18.0	1.14 (0.96-1.36)	21.3	1.05 (0.90-1.23)
	+			+			10854	23.9	1.43 (1.38-1.48)	31.5	1.34 (1.30-1.38)	2823	16.9	1.08 (1.00-1.17)	24.6	1.13 (1.06-1.21)
	+				+		16896	22.1	1.32 (1.28-1.36)	30.7	1.25 (1.22-1.28)	3174	17.3	1.11 (1.03-1.19)	24.7	1.14 (1.08-1.21)
	+					+	3978	28.2	1.66 (1.58-1.75)	36.6	1.48 (1.42-1.55)	779	22.8	1.49 (1.32-1.68)	31.1	1.43 (1.29-1.58)
		+	+				940	20.1	1.34 (1.18-1.52)	26.9	1.28 (1.16-1.42)	467	15.4	1.32 (1.08-1.61)	19.1	1.25 (1.05-1.50)
		+		+			5460	20.5	1.34 (1.27-1.41)	27.5	1.26 (1.21-1.32)	1796	14.5	1.09 (0.98-1.22)	21.2	1.14 (1.05-1.24)
		+			+		3226	17.3	1.15 (1.07-1.24)	24.0	1.13 (1.06-1.20)	1310	15.8	1.22 (1.08-1.38)	20.5	1.14 (1.03-1.27)
		+				+	898	25.6	1.65 (1.48-1.84)	32.5	1.39 (1.27-1.53)	366	21.9	1.48 (1.24-1.78)	28.1	1.42 (1.22-1.67)
			+	+			3865	21.4	1.34 (1.26-1.42)	28.2	1.31 (1.25-1.38)	2289	15.2	1.27 (1.16-1.40)	20.0	1.26 (1.17-1.36)
			+		+		798	19.4	1.34 (1.16-1.54)	24.6	1.26 (1.12-1.42)	534	18.9	1.57 (1.33-1.85)	23.0	1.51 (1.30-1.74)
			+			+	745	23.0	1.46 (1.29-1.65)	29.8	1.34 (1.20-1.49)	421	19.7	1.39 (1.17-1.65)	25.9	1.50 (1.30-1.74)
				+	+		7472	19.4	1.27 (1.21-1.33)	26.0	1.24 (1.19-1.29)	3592	16.5	1.25 (1.16-1.34)	21.8	1.23 (1.16-1.31)
				+		+	5895	29.2	1.87 (1.80-1.95)	36.9	1.71 (1.65-1.77)	2621	25.7	1.83 (1.72-1.95)	31.7	1.68 (1.59-1.77)
					+	+	672	24.9	1.67 (1.47-1.91)	31.8	1.51 (1.35-1.69)	380	24.5	1.70 (1.44-2.01)	29.5	1.56 (1.34-1.81)

Prenatal antibiotics	Smoking	Hypertension	Diabetes	Caesarean	SGA	Preterm	**Group N**	**% cases**	**Adjusted (95% CI)**	**% cases**	**Adjusted (95% CI)**	**Group N**	**% cases**	**Adjusted (95% CI)**	**% cases**	**Adjusted (95% CI)**
							Denmark					Norway				
								Follow up to 2-years age		Follow up to 10-years age			Follow up to 2-years age		Follow up to 10-years age	
+	+	+					1145	26.8	1.58 (1.44-1.74)	36.1	1.48 (1.37-1.60)	319	25.7	1.44 (1.21-1.72)	37.0	1.55 (1.34-1.80)
+	+		+				832	26.2	1.58 (1.42-1.77)	33.5	1.44 (1.31-1.59)	302	23.2	1.47 (1.20-1.79)	30.8	1.41 (1.20-1.65)
+	+			+			4873	27.2	1.63 (1.56-1.71)	35.5	1.51 (1.46-1.57)	1675	23.5	1.47 (1.36-1.60)	31.8	1.50 (1.41-1.61)
+	+				+		6920	25.2	1.47 (1.42-1.54)	33.8	1.36 (1.32-1.41)	1858	22.2	1.34 (1.24-1.45)	29.7	1.31 (1.22-1.40)
+	+					+	1665	31.8	1.97 (1.83-2.11)	41.3	1.73 (1.63-1.84)	469	31.6	1.87 (1.65-2.11)	38.4	1.69 (1.51-1.88)
+		+	+				441	21.5	1.38 (1.15-1.65)	27.2	1.35 (1.16-1.57)	229	17.9	1.23 (0.97-1.55)	23.1	1.28 (1.04-1.58)
+		+		+			2027	22.6	1.47 (1.36-1.59)	29.4	1.40 (1.30-1.49)	726	20.7	1.42 (1.24-1.63)	26.4	1.32 (1.18-1.49)
+		+			+		1068	22.3	1.45 (1.30-1.62)	29.8	1.40 (1.28-1.54)	501	18.6	1.29 (1.08-1.53)	25.9	1.40 (1.21-1.61)
+		+				+	300	28.3	1.98 (1.66-2.35)	34.3	1.73 (1.48-2.03)	149	16.8	1.36 (0.96-1.92)	23.5	1.32 (0.99-1.76)
+			+	+			1614	22.0	1.36 (1.24-1.49)	28.7	1.35 (1.25-1.46)	1007	21.8	1.57 (1.41-1.75)	26.2	1.47 (1.33-1.63)
+			+		+		304	23.4	1.43 (1.18-1.73)	28.3	1.41 (1.19-1.67)	214	17.3	1.35 (1.03-1.76)	23.8	1.57 (1.25-1.97)
+			+			+	320	31.2	2.14 (1.82-2.52)	37.8	1.92 (1.65-2.22)	216	23.6	1.70 (1.33-2.18)	31.9	1.73 (1.42-2.11)
+				+	+		2406	24.1	1.53 (1.43-1.64)	31.2	1.45 (1.37-1.54)	1330	19.2	1.40 (1.26-1.56)	24.7	1.27 (1.16-1.39)
+				+		+	2005	32.8	2.13 (2.00-2.27)	40.7	1.96 (1.86-2.07)	1100	31.9	2.08 (1.92-2.25)	37.8	1.91 (1.77-2.05)
+					+	+	214	28.0	1.76 (1.42-2.17)	38.3	1.78 (1.51-2.11)	133	23.3	1.56 (1.20-2.03)	34.6	1.57 (1.28-1.92)
	+	+	+				142	24.6	1.41 (1.06-1.86)	32.4	1.44 (1.14-1.83)					
	+	+		+			590	23.7	1.53 (1.33-1.75)	33.2	1.44 (1.29-1.62)	165	10.3	0.75 (0.46-1.20)	19.4	1.09 (0.79-1.50)
	+	+			+		575	22.8	1.38 (1.18-1.60)	28.3	1.15 (1.01-1.32)	120	14.2	1.02 (0.67-1.53)	22.5	1.09 (0.79-1.50)
	+	+				+	140	32.1	2.01 (1.58-2.56)	38.6	1.73 (1.38-2.18)					
	+		+	+			589	26.1	1.58 (1.38-1.80)	32.8	1.51 (1.34-1.69)	189	19.6	1.41 (1.08-1.85)	25.9	1.38 (1.11-1.71)
	+		+		+		207	19.3	1.34 (1.01-1.77)	27.1	1.23 (0.98-1.54)					
	+		+			+	167	32.3	1.92 (1.55-2.39)	42.5	1.66 (1.39-2.00)					
	+			+	+		2441	23.4	1.37 (1.27-1.47)	31.7	1.32 (1.24-1.40)	607	20.4	1.31 (1.13-1.51)	28.5	1.37 (1.21-1.54)
	+			+		+	1353	36.8	2.27 (2.11-2.44)	44.7	1.93 (1.81-2.06)	312	32.4	1.97 (1.69-2.28)	39.1	1.80 (1.58-2.06)
	+				+	+	397	30.2	1.69 (1.47-1.94)	38.3	1.52 (1.34-1.72)					
		+	+	+			570	20.4	1.32 (1.12-1.55)	26.8	1.32 (1.16-1.51)	306	16.0	1.24 (0.97-1.59)	22.5	1.34 (1.10-1.64)
		+	+		+		102	7.8	0.58 (0.31-1.08)	14.7	0.66 (0.43-0.99)					
		+	+			+	110	21.8	1.31 (0.94-1.82)	29.1	1.22 (0.93-1.61)					
		+		+	+		1175	20.4	1.40 (1.25-1.56)	28.8	1.35 (1.24-1.48)	419	13.1	1.01 (0.79-1.29)	17.4	1.00 (0.82-1.23)
		+		+		+	1778	28.3	1.88 (1.75-2.03)	37.2	1.74 (1.63-1.85)	822	25.8	1.67 (1.51-1.86)	31.5	1.57 (1.44-1.72)
		+			+	+	268	26.1	1.55 (1.29-1.87)	34.3	1.50 (1.28-1.76)	105	28.6	1.96 (1.46-2.62)	32.4	1.73 (1.31-2.27)

Prenatal antibiotics	Smoking	Hypertension	Diabetes	Caesarean	SGA	Preterm	**Group N**	**% cases**	**Adjusted (95% CI)**	**% cases**	**Adjusted (95% CI)**	**Group N**	**% cases**	**Adjusted (95% CI)**	**% cases**	**Adjusted (95% CI)**
							Denmark					Norway				
								Follow up to 2-years age		Follow up to 10-years age			Follow up to 2-years age		Follow up to 10-years age	
			+	+	+		223	25.1	1.55 (1.24-1.93)	30.0	1.40 (1.16-1.70)	135	14.8	1.48 (1.02-2.14)	17.0	1.33 (0.94-1.88)
			+	+		+	501	26.7	1.84 (1.60-2.12)	34.7	1.78 (1.58-2.01)	290	22.8	1.69 (1.40-2.05)	28.6	1.57 (1.33-1.84)
				+	+	+	706	30.6	2.13 (1.90-2.38)	39.4	1.87 (1.70-2.06)	437	23.8	1.83 (1.56-2.14)	30.2	1.75 (1.53-2.01)
+	+	+		+			309	30.7	1.89 (1.61-2.23)	38.8	1.76 (1.53-2.02)					
+	+	+			+		332	28.9	1.63 (1.39-1.91)	35.5	1.46 (1.26-1.68)					
+	+		+	+			319	25.7	1.57 (1.32-1.88)	34.2	1.62 (1.39-1.89)	122	28.7	1.60 (1.26-2.02)	35.2	1.49 (1.21-1.83)
+	+		+		+		104	25.0	1.63 (1.21-2.18)	27.9	1.26 (0.95-1.68)					
+	+			+	+		1049	28.4	1.72 (1.57-1.90)	36.4	1.56 (1.44-1.69)	350	23.7	1.56 (1.29-1.87)	30.9	1.53 (1.31-1.79)
+	+			+		+	624	42.5	2.43 (2.22-2.66)	51.8	2.10 (1.94-2.28)	203	30.0	1.76 (1.46-2.13)	38.9	1.71 (1.45-2.03)
+	+				+	+	190	40.5	2.29 (1.92-2.74)	46.8	1.84 (1.56-2.17)					
+		+	+	+			265	27.2	1.72 (1.43-2.07)	32.8	1.50 (1.28-1.76)	120	18.3	1.29 (0.87-1.90)	28.3	1.69 (1.25-2.29)
+		+		+	+		361	20.2	1.38 (1.12-1.69)	28.8	1.39 (1.18-1.64)	150	14.7	0.90 (0.63-1.29)	20.7	1.05 (0.79-1.40)
+		+		+		+	644	35.6	2.37 (2.14-2.63)	44.9	2.17 (1.99-2.37)	303	30.0	2.13 (1.81-2.51)	35.0	1.83 (1.57-2.13)
+			+	+		+	251	34.3	2.36 (2.02-2.77)	39.8	2.14 (1.85-2.47)	150	30.0	1.85 (1.48-2.33)	36.0	1.89 (1.54-2.31)
+				+	+	+	229	41.5	2.30 (1.98-2.66)	50.2	2.07 (1.83-2.35)	156	32.7	1.94 (1.59-2.37)	42.9	2.06 (1.74-2.45)
	+	+		+	+		238	22.7	1.47 (1.16-1.86)	30.7	1.33 (1.10-1.62)					
	+	+		+		+	195	32.3	2.00 (1.65-2.43)	40.5	1.67 (1.42-1.96)					
	+			+	+	+	390	34.4	2.13 (1.86-2.44)	45.6	1.96 (1.75-2.19)					
		+	+	+		+	308	29.5	1.95 (1.65-2.30)	36.4	1.68 (1.45-1.94)	131	16.8	1.25 (0.90-1.74)	18.3	1.03 (0.74-1.43)
		+		+	+	+	848	26.5	1.77 (1.59-1.97)	35.4	1.60 (1.46-1.74)	281	26.0	1.97 (1.64-2.37)	33.5	1.85 (1.58-2.17)
+	+	+		+	+		107	30.8	1.73 (1.29-2.30)	34.6	1.31 (1.01-1.70)					
+	+			+	+	+	185	36.2	2.62 (2.14-3.20)	48.1	2.29 (1.94-2.70)					
+		+	+	+		+	135	27.4	1.98 (1.52-2.58)	37.0	1.80 (1.46-2.22)					
+		+		+	+	+	263	29.7	1.97 (1.64-2.38)	35.4	1.79 (1.52-2.12)	117	32.5	2.28 (1.77-2.94)	43.6	2.16 (1.75-2.67)
	+	+		+	+	+	178	35.4	2.37 (1.92-2.92)	43.8	1.82 (1.52-2.18)					
All other combinations							1526	30.2	1.90 (1.76-2.04)	36.8	1.68 (1.57-1.79)	1581	22.9	1.61 (1.47-1.75)	29.7	1.54 (1.43-1.66)

Supplementary Table 5 – Full results for interaction analyses

Pairwise Exposure Combination	Hazard ratio or Interaction Term (95% CI) Denmark	Hazard ratio or Interaction Term (95% CI) Norway
Prenatal antibiotics=0 Smoking=0	Reference	Reference
Prenatal antibiotics=0 Smoking=1	1.18 (1.17 to 1.19)	1.06 (1.04 to 1.09)
Prenatal antibiotics=1 Smoking=0	1.18 (1.17 to 1.19)	1.20 (1.18 to 1.21)
Prenatal antibiotics=1 Smoking=1	1.35 (1.34 to 1.37)	1.30 (1.27 to 1.33)
Multiplicative interaction term	0.98 (0.96 to 0.99)	1.02 (0.99 to 1.05)
Additive interaction term	-0.002 (-0.024 to 0.020)	0.037 (-0.002 to 0.076)
Prenatal antibiotics=0 Hypertension=0	Reference	Reference
Prenatal antibiotics=0 Hypertension=1	1.18 (1.16 to 1.20)	1.14 (1.10 to 1.17)
Prenatal antibiotics=1 Hypertension=0	1.18 (1.17 to 1.19)	1.21 (1.19 to 1.22)
Prenatal antibiotics=1 Hypertension=1	1.35 (1.32 to 1.39)	1.32 (1.27 to 1.37)
Multiplicative interaction term	0.97 (0.94 to 1.00)	0.96 (0.91 to 1.01)
Additive interaction term	-0.006 (-0.046 to 0.034)	-0.025 (-0.088 to 0.039)
Prenatal antibiotics=0 Diabetes=0	Reference	Reference
Prenatal antibiotics=0 Diabetes=1	1.15 (1.12 to 1.18)	1.18 (1.14 to 1.22)
Prenatal antibiotics=1 Diabetes=0	1.18 (1.17 to 1.19)	1.20 (1.19 to 1.22)
Prenatal antibiotics=1 Diabetes=1	1.32 (1.28 to 1.36)	1.40 (1.35 to 1.46)
Multiplicative interaction term	0.98 (0.94 to 1.02)	0.98 (0.93 to 1.04)
Additive interaction term	-0.006 (-0.055 to 0.044)	0.015 (-0.055 to 0.085)
Prenatal antibiotics=0 Caesarean section=0	Reference	Reference
Prenatal antibiotics=0 Caesarean section=1	1.22 (1.21 to 1.23)	1.17 (1.15 to 1.19)
Prenatal antibiotics=1 Caesarean section=0	1.18 (1.17 to 1.19)	1.21 (1.19 to 1.22)
Prenatal antibiotics=1 Caesarean section=1	1.42 (1.40 to 1.44)	1.38 (1.35 to 1.41)
Multiplicative interaction term	0.99 (0.97 to 1.01)	0.98 (0.95 to 1.01)
Additive interaction term	0.022 (-0.002 to 0.046)	0.006 (-0.031 to 0.043)
Prenatal antibiotics=0 SGA=0	Reference	Reference
Prenatal antibiotics=0 SGA=1	1.10 (1.08 to 1.11)	1.09 (1.07 to 1.11)
Prenatal antibiotics=1 SGA=0	1.18 (1.17 to 1.19)	1.21 (1.20 to 1.23)
Prenatal antibiotics=1 SGA=1	1.26 (1.24 to 1.29)	1.26 (1.22 to 1.29)
Multiplicative interaction term	0.98 (0.96 to 1.00)	0.95 (0.92 to 0.98)
Additive interaction term	-0.011 (-0.038 to 0.018)	-0.048 (-0.090 to -0.005)
Prenatal antibiotics=0 Preterm=0	Reference	Reference
Prenatal antibiotics=0 Preterm=1	1.47 (1.44 to 1.49)	1.42 (1.39 to 1.46)
Prenatal antibiotics=1 Preterm=0	1.18 (1.17 to 1.18)	1.21 (1.19 to 1.22)
Prenatal antibiotics=1 Preterm=1	1.72 (1.68 to 1.76)	1.64 (1.58 to 1.69)
Multiplicative interaction term	1.00 (0.97 to 1.03)	0.95 (0.91 to 0.99)
Additive interaction term	0.082 (0.036 to 0.128)	0.005 (-0.060 to 0.070)
Smoking=0 Hypertension=0	Reference	Reference
Smoking=0 Hypertension=1	1.19 (1.18 to 1.21)	1.14 (1.11 to 1.17)
Smoking=1 Hypertension=0	1.19 (1.18 to 1.2)	1.10 (1.08 to 1.11)
Smoking=1 Hypertension=1	1.34 (1.29 to 1.38)	1.17 (1.09 to 1.26)
Multiplicative interaction term	0.94 (0.91 to 0.98)	0.94 (0.87 to 1.02)
Additive interaction term	-0.046 (-0.095 to 0.004)	-0.061 (-0.153 to 0.031)
Smoking=0 Diabetes=0	Reference	Reference
Smoking=0 Diabetes=1	1.16 (1.14 to 1.18)	1.20 (1.17 to 1.24)
Smoking=1 Diabetes=0	1.18 (1.17 to 1.19)	1.10 (1.08 to 1.11)
Smoking=1 Diabetes=1	1.30 (1.25 to 1.36)	1.16 (1.07 to 1.25)
Multiplicative interaction term	0.95 (0.91 to 1.00)	0.88 (0.81 to 0.96)
Additive interaction term	-0.040 (-0.010 to 0.021)	-0.141 (-0.241 to -0.041)
Smoking=0 Caesarean section=0	Reference	Reference

Smoking=0 Caesarean section=1	1.22 (1.21 to 1.23)	1.16 (1.15 to 1.18)
Smoking=1 Caesarean section=0	1.18 (1.17 to 1.19)	1.09 (1.07 to 1.11)
Smoking=1 Caesarean section=1	1.42 (1.40 to 1.45)	1.26 (1.22 to 1.31)
Multiplicative interaction term	0.99 (0.97 to 1.01)	1.00 (0.96 to 1.04)
Additive interaction term	0.023 (-0.007 to 0.052)	0.012 (-0.039 to 0.063)
Smoking=0 SGA=0	Reference	Reference
Smoking=0 SGA=1	1.08 (1.06 to 1.09)	1.07 (1.05 to 1.09)
Smoking=1 SGA=0	1.18 (1.17 to 1.19)	1.09 (1.07 to 1.11)
Smoking=1 SGA=1	1.23 (1.21 to 1.26)	1.15 (1.11 to 1.19)
Multiplicative interaction term	0.97 (0.95 to 0.99)	0.99 (0.95 to 1.03)
Additive interaction term	-0.024 (-0.051 to 0.003)	-0.007 (-0.057 to 0.044)
Smoking=0 Preterm=0	Reference	Reference
Smoking=0 Preterm=1	1.48 (1.46 to 1.5)	1.41 (1.38 to 1.44)
Smoking=1 Preterm=0	1.18 (1.17 to 1.19)	1.09 (1.07 to 1.11)
Smoking=1 Preterm=1	1.65 (1.60 to 1.69)	1.48 (1.41 to 1.56)
Multiplicative interaction term	0.94 (0.92 to 0.97)	0.97 (0.91 to 1.02)
Additive interaction term	-0.013 (-0.060 to 0.034)	-0.016 (-0.101 to 0.068)
Hypertension=0 Diabetes=0	Reference	Reference
Hypertension=0 Diabetes=1	1.15 (1.12 to 1.17)	1.19 (1.16 to 1.23)
Hypertension=1 Diabetes=0	1.18 (1.16 to 1.19)	1.13 (1.10 to 1.16)
Hypertension=1 Diabetes=1	1.26 (1.20 to 1.33)	1.22 (1.12 to 1.32)
Multiplicative interaction term	0.94 (0.89 to 0.99)	0.90 (0.83 to 0.99)
Additive interaction term	-0.060 (-0.130 to 0.009)	-0.103 (-0.213 to 0.006)
Hypertension=0 Caesarean section=0	Reference	Reference
Hypertension=0 Caesarean section=1	1.21 (1.20 to 1.22)	1.16 (1.15 to 1.18)
Hypertension=1 Caesarean section=0	1.15 (1.13 to 1.17)	1.12 (1.08 to 1.15)
Hypertension=1 Caesarean section=1	1.38 (1.35 to 1.42)	1.26 (1.21 to 1.31)
Multiplicative interaction term	0.99 (0.96 to 1.02)	0.97 (0.92 to 1.02)
Additive interaction term	0.023 (-0.016 to 0.063)	-0.021 (-0.085 to 0.042)
Hypertension=0 SGA=0	Reference	Reference
Hypertension=0 SGA=1	1.09 (1.08 to 1.1)	1.07 (1.05 to 1.09)
Hypertension=1 SGA=0	1.19 (1.17 to 1.2)	1.12 (1.09 to 1.15)
Hypertension=1 SGA=1	1.21 (1.17 to 1.25)	1.20 (1.14 to 1.27)
Multiplicative interaction term	0.93 (0.90 to 0.97)	1.00 (0.94 to 1.07)
Additive interaction term	-0.069 (-0.115 to -0.024)	0.008 (-0.069 to 0.084)
Hypertension=0 Preterm=0	Reference	Reference
Hypertension=0 Preterm=1	1.46 (1.44 to 1.48)	1.40 (1.37 to 1.43)
Hypertension=1 Preterm=0	1.14 (1.13 to 1.16)	1.09 (1.06 to 1.12)
Hypertension=1 Preterm=1	1.60 (1.54 to 1.65)	1.46 (1.39 to 1.54)
Multiplicative interaction term	0.96 (0.92 to 0.99)	0.95 (0.90 to 1.02)
Additive interaction term	-0.008 (-0.066 to 0.050)	-0.032 (-0.119 to 0.055)
Diabetes=0 Caesarean section=0	Reference	Reference
Diabetes=0 Caesarean section=1	1.22 (1.20 to 1.23)	1.16 (1.15 to 1.18)
Diabetes=1 Caesarean section=0	1.13 (1.10 to 1.16)	1.19 (1.16 to 1.23)
Diabetes=1 Caesarean section=1	1.35 (1.30 to 1.39)	1.29 (1.23 to 1.35)
Multiplicative interaction term	0.98 (0.94 to 1.02)	0.93 (0.88 to 0.98)
Additive interaction term	-0.0001 (-0.050 to 0.049)	-0.068 (-0.140 to 0.004)
Diabetes=0 SGA=0	Reference	Reference
Diabetes=0 SGA=1	1.09 (1.08 to 1.10)	1.08 (1.06 to 1.09)
Diabetes=1 SGA=0	1.16 (1.13 to 1.18)	1.19 (1.16 to 1.22)
Diabetes=1 SGA=1	1.22 (1.14 to 1.3)	1.35 (1.23 to 1.48)
Multiplicative interaction term	0.97 (0.90 to 1.04)	1.06 (0.95 to 1.17)
Additive interaction term	-0.028 (-0.112 to 0.057)	0.085 (-0.053 to 0.222)

Diabetes=0 Preterm=0	Reference	Reference
Diabetes=0 Preterm=1	1.47 (1.45 to 1.49)	1.41 (1.38 to 1.44)
Diabetes=1 Preterm=0	1.13 (1.11 to 1.15)	1.18 (1.15 to 1.22)
Diabetes=1 Preterm=1	1.54 (1.47 to 1.62)	1.46 (1.36 to 1.57)
Multiplicative interaction term	0.93 (0.88 to 0.98)	0.88 (0.81 to 0.95)
Additive interaction term	-0.056 (-0.136 to 0.024)	-0.133 (-0.246 to -0.019)
Caesarean section=0 SGA=0	Reference	Reference
Caesarean section=0 SGA=1	1.09 (1.08 to 1.10)	1.07 (1.05 to 1.09)
Caesarean section=1 SGA=0	1.22 (1.21 to 1.23)	1.16 (1.14 to 1.18)
Caesarean section=1 SGA=1	1.32 (1.29 to 1.35)	1.26 (1.22 to 1.31)
Multiplicative interaction term	0.99 (0.97 to 1.02)	1.02 (0.98 to 1.06)
Additive interaction term	0.011 (-0.022 to 0.045)	0.034 (-0.018 to 0.086)
Caesarean section=0 Preterm=0	Reference	Reference
Caesarean section=0 Preterm=1	1.38 (1.36 to 1.41)	1.33 (1.30 to 1.37)
Caesarean section=1 Preterm=0	1.17 (1.16 to 1.19)	1.12 (1.11 to 1.14)
Caesarean section=1 Preterm=1	1.74 (1.7 to 1.77)	1.60 (1.56 to 1.65)
Multiplicative interaction term	1.07 (1.04 to 1.10)	1.07 (1.03 to 1.12)
Additive interaction term	0.183 (0.141 to 0.224)	0.152 (0.091 to 0.214)
SGA=0 preterm=0	Reference	Reference
SGA=0 preterm=1	1.47 (1.45 to 1.49)	1.39 (1.36 to 1.42)
SGA=1 preterm=0	1.09 (1.08 to 1.1)	1.07 (1.05 to 1.09)
SGA=1 preterm=1	1.64 (1.58 to 1.7)	1.64 (1.54 to 1.73)
Multiplicative interaction term	1.03 (0.99 to 1.07)	1.10 (1.03 to 1.17)
Additive interaction term	0.085 (0.021 to 0.149)	0.176 (0.076 to 0.276)
